# Crohn’s disease in a developing African mission hospital: a case report

**DOI:** 10.1186/s13256-019-1971-5

**Published:** 2019-03-07

**Authors:** Bamidele Johnson Alegbeleye

**Affiliations:** Department of Surgery, St Elizabeth Catholic General Hospital, Shisong, P.O Box 8, Kumbo - Nso, Bui Division, Northwestern Region Cameroon

**Keywords:** Crohn’s disease, Autoimmune disorder, Granulomatous colitis, Regional enteritis

## Abstract

**Background:**

A case is reported of innocuous intestinal obstruction requiring surgical intervention that was confirmed to be Crohn’s disease histopathologically in a resource-constrained rural mission hospital in Cameroon.

**Case presentation:**

A 70-year man of Kumbo origin from Northwest region of Cameroon with a history of crampy right lower-quadrant abdominal pain, non-bloody, non-mucoid diarrhea alternating with constipation presented to my institution. Abdominal examination of the patient revealed an ill-defined mass in the right iliac fossa and visible peristalsis. An abdominal computed tomographic scan and barium enema study confirmed a complex ascending colonic and cecal tumor. The patient underwent exploratory laparotomy. The intraoperative finding was a huge complex inflammatory mass involving the cecum, terminal ileum, and sigmoid colon. He subsequently had sigmoidectomy with end–to-end sigmoidorectal anastomosis and a cecal resection, and the proximal ascending colon was exteriorized because end mucoid fistula and terminal ileostomy were performed. The histopathological diagnosis confirmed Crohn’s disease. The patient subsequently received five courses of adjuvant chemotherapy consisting of azathioprine, methotrexate, mesalamine, and methylprednisolone. He had complete disease remission and subsequently had closure of ileostomy with satisfactory postoperative status. The most recent follow-up abdominal computed tomographic scan and colonoscopy revealed disease-free status. The patient is also currently receiving a maintenance dose of rectal mesalamine and oral omeprazole treatment. He has been followed every 2 months in the surgical outpatient clinic over the last 16 months with satisfactory clinical outcome.

**Conclusions:**

Crohn’s disease is uncommon in Africa, and this entity is encountered sparingly. The signs and symptoms of Crohn’s disease overlap with many other abdominal disorders, such as tuberculosis, ulcerative colitis, irritable bowel syndrome, and others. Several publications in the literature describe that it is difficult to make an accurate diagnosis of this disease, despite the fact that many diagnostic armamentaria are available to suggest its presence. Most of the patients with Crohn’s disease are treated conservatively, and a few may require surgical intervention, especially those presenting with complications such as intestinal obstruction, perforations, and abscess as well as fistula formations, as seen in this index patient. Crohn’s disease is considered by many to be a very rare disease in Africa. It is interesting to know that Crohn’s disease, which affects mainly young adults, may debut at any age. The rarity and clinical curiosity of this entity suggested reporting of my patient’s case. Evidence-based up-to-date information on Crohn’s disease is also documented.

## Introduction

Crohn’s disease (CD), also referred to as regional enteritis, granulomatous enterocolitis, and terminal ileitis, is a chronic relapsing and remitting inflammatory disease of unknown cause that is often multifocal and can affect any portion of the gastrointestinal tract (GIT) [1]. It is generally accepted that the Scottish surgeon Dalziel gave the first account of the disease in 1913 [2]. In the 1960s, Lockhart-Mummery and Morson described the involvement of the large intestine by CD [3], and it was in the 1950s that anal and anorectal CD were described [4]. In time, it became clear that CD can involve any part of the digestive tract and that extraintestinal manifestations can be present, especially in the skin, eyes, and joints [5–9].

Epidemiological studies showed that CD has an incidence of 3 to 20 cases per 100,000 [10, 11]. CD is more common in the industrialized world, particularly in North America and Western Europe, though the incidence is rising in Asia and South America [10, 12, 13]. There may be a slightly higher predominance of CD in women, and it is more common in individuals of Ashkenazi Jewish origin than in non-Jews. The exact pathogenesis of CD is unknown, although there are a number of genetic and environmental factors that have been shown to increase the risk of the disease and lead to the aberrant gut immune response characteristic of the disease [10, 12].

In a related development, other literature suggested a “north to south gradient” with respect to the incidence of CD. North America (7 to 10.3/100,000 per year), the United Kingdom (8.3 to 9.1/100,000 per year), and Northern Europe (5.8–6.3/100,000 per year) have the highest incidence of CD [10, 12, 13]. The prevalence of CD also demonstrated a similar pattern, which is reported to be 207/100,000 per year in North America, 156/100,000 per year in the United Kingdom, and 90/100,000 per year in Northern Europe [14–20]. However, owing to limited information on inflammatory bowel disease (IBD) in southern countries, this “gradient” is not widely accepted.

Furthermore, there are only a few studies on the incidence and prevalence of CD in Latin America [14, 21]. Although the incidence of CD in South America has been surveyed to be lower than in North America, in the last 50 years, occidental countries have reported a rise in both incidence and prevalence of IBD [14, 21]. Another study mentioned that Puerto Rico has the lowest incidence of IBD within the southern American subregion [14, 22]. Yamamoto-Furusho, in his study of a Mexican population, reported an increasing incidence of ulcerative colitis (UC) from 1987 to 2006, with a 2.6-fold increase from 1997 to 2006, compared with the previous decade. This increasing incidence of UC has been linked to environmental factors and the unique genetic mosaic of the Mexican population, but there is no information regarding CD incidence in Mexico to date [14, 23].

The rarity of CD and the prevalence of tuberculous enterocolitis in Africa and Asia in general tend to unnecessarily make clinicians hesitant to diagnose a CD both clinically and histologically [9, 24–27]. Its differentiation from UC is always a problem, but the distinction from tuberculosis is rather more difficult in developing countries if the lesion is granulomatous [9, 24–27]. Surgically resected bowel segments again require a close examination using various diagnostic criteria to diagnose or exclude a CD [9, 24–27]. This case of histopathologically confirmed CD is presented because of the rarity and clinical curiosity of this entity. Evidence-based, up-to-date information on CD is also presented.

## Case presentation

**Fig. 1:**
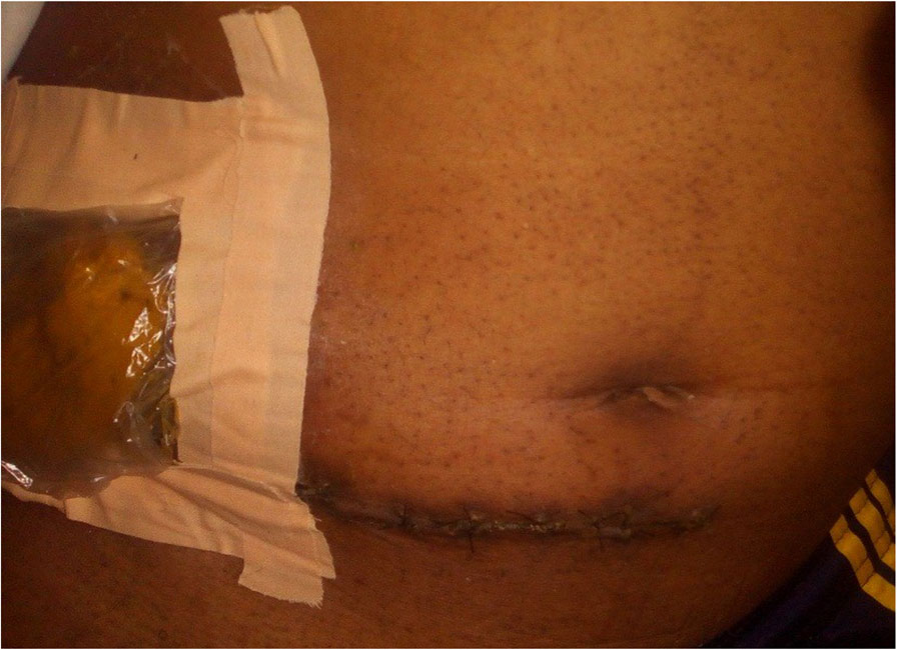
The index patient

**Fig. 2:**
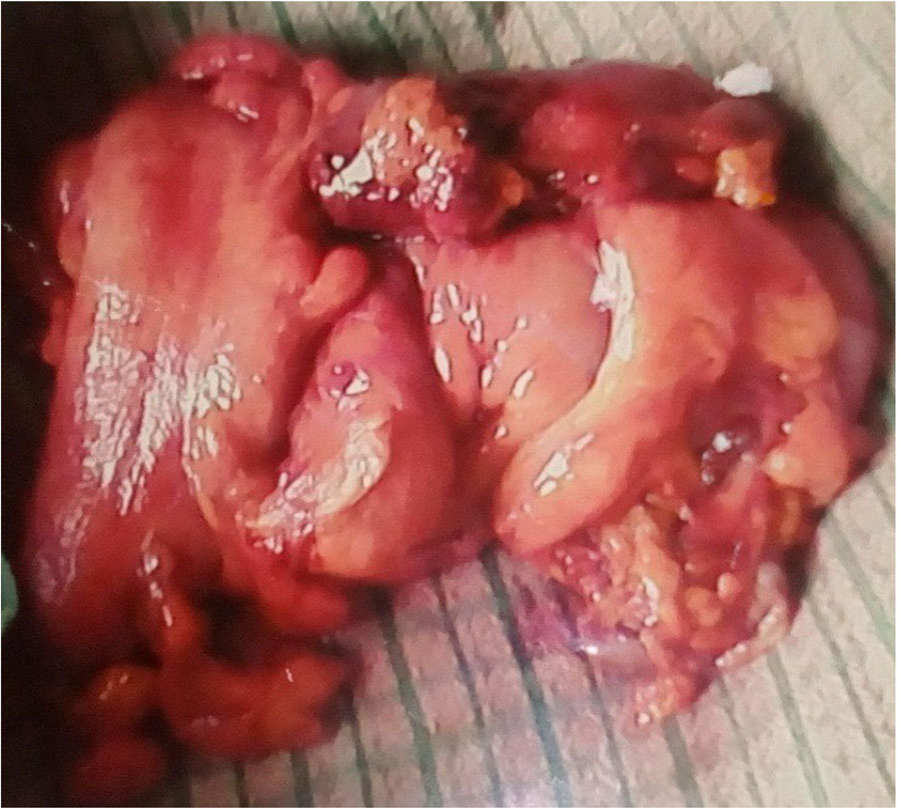
Post op bowel specimen

**Fig. 3:**
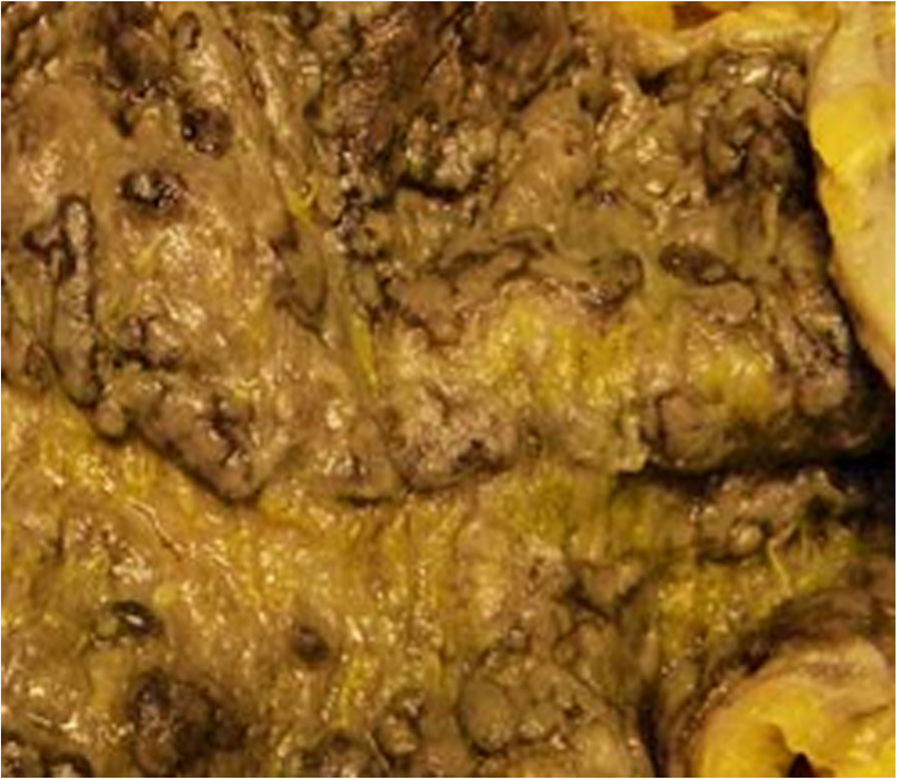
Colonic Crohn’s disease

**Fig. 4:**
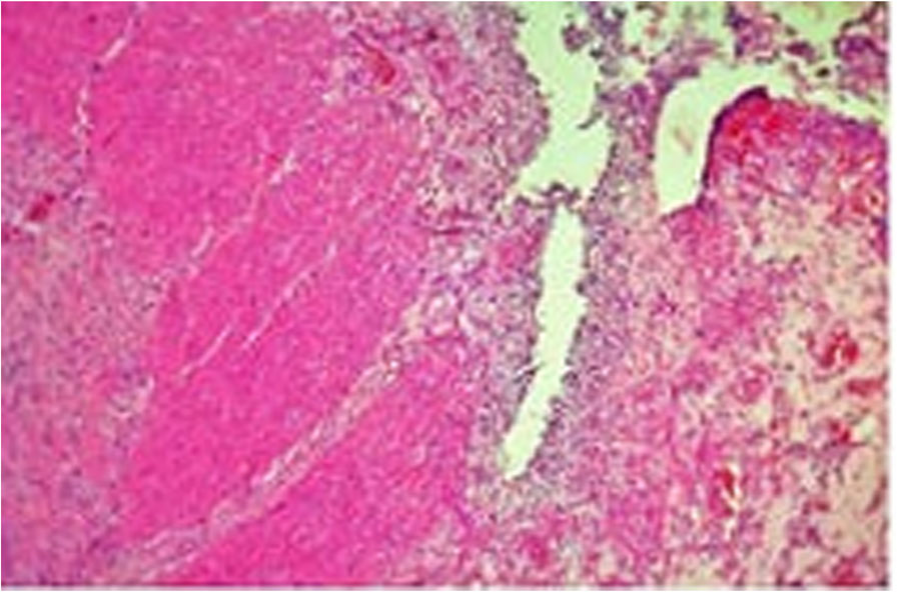
The histopathology slide

A 70-year-old man of Kumbo origin from the Northwest region of Cameroon was admitted to a rural mission hospital in Cameroon with a history of crampy right lower-quadrant abdominal pain, nonbloody, nonmucoid diarrhea alternating with constipation for the last 5 days. Anorexia and low-grade fever were observed, but no weight loss. Abdominal examination revealed the features of acute intestinal obstruction with an ill-defined mass in the right iliac fossa (RIF) and visible peristalsis. A chest radiograph was essentially normal. An abdominopelvic ultrasound scan showed dilated bowel loops and an RIF mass. An abdominal plain radiograph showed multiple air-fluid levels and dilated bowel loops. An abdominal computed tomographic (CT) scan and barium enema study confirmed a complex ascending colonic and cecal tumor. The patient had a markedly raised white blood cell count of 40,300 cells/ml. The C-reactive protein was significantly elevated, and results of the tuberculin test and Genexpert test for tuberculosis were both negative. The patient’s blood pressure was 129/78 mmHg, and his pulse rate was 60 beats/min. He also had pyrexia (− 37.9 °C). In view of acute intestinal obstruction, exploratory laparotomy was performed after routine investigations. The intraoperative finding was a huge complex inflammatory mass involving the cecum, terminal ileum, and sigmoid colon. The patient subsequently had a sigmoidectomy with end-to-end sigmoidorectal anastomosis and a cecal resection, and the proximal ascending colon was exteriorized because end mucoid fistula and terminal ileostomy were performed. Figure 1 is a postoperative photograph showing the ileostomy and disposable stoma bag. Figure 2 is the immediate postoperative photograph of the resected complex mass involving the cecum, terminal ileum, and sigmoid colon. Histopathological examination of the resected specimen showed macroscopic appearance of a complex large mass (Fig. 3) involving the cecum, sigmoid colon, and terminal ileum, with congested swollen mucosa interspersed with diffused, irregular ulcerations with a cobblestone appearance (noncaseating granulomas in all layers of bowel wall from serosa to mucosa). Also, multiple fissures were present, as well as perforation and fistula seen between adjourning bowel loops, but no significant IBD was observed in nonulcerated mucosa. Microscopically, prominent and enlarged lymphatic follicles, proliferation of muscularis mucosa, and formation of fissures extending from mucosa to serosa along with gross edema. Marked infiltrates of inflammatory cells involved all the bowel layers; the details are depicted in Fig. 4. Therefore, histopathological diagnosis of CD was made. The postoperative period was uneventful. Thereafter, the patient received five courses of adjuvant chemotherapy consisting of azathioprine (AZA), methotrexate (MTX), mesalamine, and methylprednisolone. He had complete disease remission and subsequently underwent closure of the ileostomy with satisfactory postoperative status. The most recent follow-up abdominal CT scan and colonoscopy revealed disease-free status. The patient is also currently receiving a maintenance dose of rectal mesalamine and oral omeprazole treatment. He has been followed every 2 months in the surgical outpatient clinic over the last 16 months with a satisfactory clinical outcome.

## Discussion

CD causes inflammation of the digestive tract. It can affect any area of the GIT from mouth to anus; however, it most commonly affects the ileum [28]. In CD, all layers of the intestine may be involved, and normal healthy bowel can be found between sections of diseased bowel. CD affects men and women equally in all age groups, with a predilection in the second and third decades and familial preponderance in a few [29]. IBD was once considered rare in the developing world; its epidemiology is changing, and the incidence of both CD and UC is increasing in the Asia-Pacific region, India, Eastern Europe, and South Africa [30–33]. There have been very few reported cases of CD in black African patients in Kenya and South Africa [34]. The symptoms, anatomical distribution, signs, and pathology in black patients in Africa and America are similar to those described in white individuals. The disease is probably underdiagnosed in Africa because of difficulties in distinguishing schistosomiasis and tuberculosis of the bowel [24, 35–37]. The etiology of CD remains incompletely known, although several theories have been issued, such as the involvement of genetic factors, environmental factors (including diet), and infective agents [24–26]. CD is directly correlated with a triad of predisposing factors, including genetic problems, immune system malfunctions, and environmental factors [24–26].

CD usually presents with abdominal pain especially due to involvement of ileum, blood-stained diarrhea, and anemia. Some patients with CD may have low-grade fever, nausea, and vomiting. Fissures or cracks may be evident, and fistulas and abscesses may form in anal involvement [38]. CD may also present with extraintestinal manifestations such as skin or mouth lesions, pain in the joints, eye irritation, kidney stones, gallstones, and other diseases of the hepatobiliary system [39]. Affected children may have delayed milestones. Patients with severe cases of CD may have most common complications such as intestinal blockage with thickening and fibrosis of the affected segment [40].

The diagnosis of CD is a clinical one and can be quite difficult, given that the presenting symptoms can be insidious and nonspecific [41]. Red flag symptoms that require further evaluation include weight loss, bloody diarrhea, iron deficiency, and nighttime awakenings.

Similarly, significant family history of IBD, unexplained elevations in the C-reactive protein level and erythrocyte sedimentation rate, or other acute-phase reactants (for example, ferritin and platelets), or low vitamin B_12_ should prompt further investigation for possible CD. Laboratory findings that are useful in CD are hypoalbuminemia, elevation of the erythrocyte sedimentation rate and C-reactive protein, anemia, or leukocytosis [41–43]. The serologic markers of clinical importance are the anti-*Saccharomyces cerevisiae* antibodies, which are commonly positive in CD, and antineutrophil cytoplasmic antibody, which is negative for CD [41–43]. These tests are suggestive of CD but are not meant to be interpreted as diagnostic tests, because positive results could be present in a healthy population The main utility of these antibodies is for differential diagnosis in patients with characteristics of CD and other diseases, including UC [41–43].

In spite of the widely used diagnostic modalities such as ultrasound, barium x-rays, CT scans, and colonoscopy, a clear diagnosis of CD may remain obscure. Although no single “gold standard” indicator of this disease has been established, it is indeed possible to make an ideal diagnosis based on the patient’s clinical, laboratory, endoscopic, and pathologic data; meanwhile, colonoscopy, capsular endoscopy, and laparoscopy significantly assist clinicians worldwide in elucidating the diagnosis [42]. Both computed tomography enterography (CTE) and magnetic resonance enterography (MRE) allow visualization of the bowel wall, mucosa, and extraluminal complications. CTE and MRE have supplanted small-bowel barium studies as the criterion standard for the diagnosis and assessment of CD [14, 43].

Treatment and prognosis of the disease depend on several factors. The Montreal classification considers age of onset, location, and behavior of the disease, as well as presence of perianal disease, for categorization; many decisions regarding diagnostic approach, treatment, follow-up, and prediction of several outcomes, ranging from response to therapy to long-term prognosis, depend on this classification (Table 1) [44].

**Table 1:** Montreal classification [10]

Age at diagnosis	A1: less than 16 years
	A2: between 17 and 40 years
	A3: over 40 years
Location	L1: ileal
	L2: colonic
	L3: ileocolonic
	L4: isolated upper digestive
Behavior	B1: nonstricturing, nonpenetrating
	B2: stricturing
	B3: penetrating
	P: perianal disease

Furthermore, for severity scoring, there is an extensive number of validated scores such as the Crohn’s Disease Activity Index (CDAI), mainly used in clinical trials because of its complexity, and the Harvey-Bradshaw Index used in the clinical setting owing to its simplicity (Table 2) [45–47].

**Table 2:** Crohn’s Disease Activity Index [14]

Variable	Variable description	Multiply	Total
1	No. of liquid or soft stools (each day for 7 days)	×2	
2	Abdominal pain, sum of seven daily ratings (0 = none, 1 = mild, 2 = moderate, 3 = severe)	×5	
3	Abdominal mass (0 = no, 2 = questionable, 5 = definite)	×10	
4	General well-being, sum of seven daily ratings (0 = generally well, 1 = slightly under par)	×7	
5	Hematocrit (males 47%, females 42%)	×6	
6	Body weight (1-weight/standard weight) (add or subtract according to sign)	×100	
7	Use of diphenoxylate or loperamide for diarrhea (0 = no, 1 = yes)	×30	
8	Number of listed complications (arthritis or arthralgia, iritis or uveitis, erythema nodosum or pyoderma gangrenosum or aphthous stomatitis, anal fissure or fistula or abscess, other fistula, fever over 37.8 °C).	×20	

*Note*: Add the eight variables. A total of < 150 points denotes disease remission and a better outcome; > 450 points implies severe disease

There is a global resolve among clinicians that the treatment of CD should depend on disease severity, location of disease, and subtype of disease (that is, inflammatory, stricturing, or penetrating). Attempts are also being made to determine individuals who are at risk for aggressive CD and who may require earlier and more aggressive therapies. Risk factors for aggressive disease activity include age of diagnosis less than 30 years, extensive anatomic involvement, perianal disease, deep ulcers, prior surgery, and stricturing and/or penetrating disease [10, 48]. One of the biggest challenges associated with CD is that after 20 years of disease activity, 80% of patients will require surgery, and approximately 30% will require surgery within 5 years of diagnosis [10, 41, 48]. Although the goal of medical therapy is to maintain remission without the need for surgery, once strictures and/or fistula complications occur, surgery may be required. Unfortunately, because surgery is not curative for CD, many patients will require multiple surgeries over their lifetime [10, 49].

There are a number of different drugs used to treat CD, as highlighted in Table 3. Mesalamine has been evaluated in a number of studies but has not been shown to effectively induce or maintain remission in CD. The perceived benefit of mesalamine is likely related to its safety profile [10, 41]. Antibiotics are also used in CD, but the evidence supporting their use is also limited [50]. The main role of antibiotics is to treat the suppurative or perianal complications of CD [51].

**Table 3:** Medications, monitoring, and adverse events ^a^ [10]

Group	Drugs	Routes	Efficacy	Recommended testing	Adverse drug reactions
5-Aminosalycilates	Mesalamine Balsalazide Sulfasalazine	Oral Rectal	Induction and maintenance	Cr, urinalysis CBC, LFTs	Headache, nausea, diarrhea Paradoxical worsening of symptoms Interstitial nephritis Hemolytic anemia,^a^ leukopenia,^a^ hepatitis^a^
Corticosteroids	Prednisone Budesonide Methylprednisolone	Oral Rectal IV	Induction only	Consider checking hemoglobin A1c and vitamin D levels If prolonged steroid > 3 mo: DEXA scan and ophthalmologic evaluation	Osteopenia/osteoporosis Avascular necrosis Serious infection Weight gain Insomnia Mood changes Delirium Cataracts Glaucoma Skin changes Delayed wound healing Adrenal insufficiency
Thiopurines	Azathioprine Mercaptopurine	Oral	Maintenance	TPMT enzyme activity or genetics before initiation CBC, LFTs Skin examinations Yearly Pap smear in women	Nausea Vomiting Transaminitis Bone marrow suppression Pancreatitis Infection Non-Hodgkin lymphoma Nonmelanoma skin cancer Cervical dysplasia
Methotrexate^b^	Methotrexate	SC or IM (limited efficacy of oral route)	Induction and maintenance	CBC, LFTs	Infection Cytopenias Transaminitis Cirrhosis Nausea/vomiting Flulike symptoms Oral ulcers Pulmonary toxicity Contraindicated in pregnancy
Anti-TNF	Infliximab Adalimumab Certolizumab pegol	IV SC	Induction and maintenance	Latent TB and hepatitis B before initiation CBC, LFTs Skin examinations	Infusion/injection site reaction Infection Non-Hodgkin lymphoma (mostly if combined with a thiopurine) HSTC-L (if combined with a thiopurine) Melanoma Reactivation of latent TB and hepatitis B Drug-induced lupus erythematosus Demyelinating disease Psoriasiform and eczema reactions Worsening of CHF
Adhesion molecule inhibitors	Natalizumab Vedolizumab	IV	Induction and maintenance	Natalizumab: JC virus checking before initiation and yearly monitoring for JC virus Vedolizumab and natalizumab: Consider CBC	Infusion reactions Infection Nasopharyngeal polyps PML (natalizumab only with positive JC virus
IL-12/IL-23 inhibitors	Ustekinumab	IV SC	Induction and maintenance	Latent TB before initiation Consider CBC, LFTs	Infusion reactions Skin cancer Reversible posterior leukoencephalopathy TB

*Abbreviations: Anti-TNF* Anti-tumor necrosis factor, *CBC* Complete blood cell count, *Cr* Creatinine, *CHF* Congestive heart failure, *DEXA* Dual-energy X-ray absorptiometry, *IV* Intravenous, *IM* Intramuscular, *SC* Subcutaneous, *JC* John Cunningham, *LFT* Liver function test, *FDA* Food and Drug Administration, *HSTLC* Hepatosplenic T-cell lymphoma, *PML* Progressive multifocal leukoencephalopathy, *Pap* Papanicolaou, *TB* Tuberculosis, *TPMT* Thiopurine methyltransferase

^a^Sulfasalazine only

^b^Patients should be given 1 g of folic acid with the medication to reduce side effects

Immunosuppressants, including AZA, mecaptopurine (MP), and MTX, have been used for many years to treat CD. These drugs are typically used to maintain remission because of their slow onset of action. However, more recent studies question the overall efficacy of AZA/MP as monotherapy and their use in early CD [10, 52–54]. Newer reports have suggested that these drugs can be used in combination with anti-tumor necrosis factor (anti-TNF) drugs to decrease their immunogenicity and also to increase anti-TNF drug concentrations. The mainstay of therapy for CD has been anti-TNF agents.

More recently approved drugs are monoclonal antibodies directed against certain integrins (α4 or α4β7) or interleukins (IL-12/IL-23). The first anti-integrin approved for CD was natalizumab, but this is associated with progressive multifocal leukoencephalopathy (PML), a fatal brain infection [10, 55, 56]. Vedolizumab is a gut-selective anti-integrin that has not been associated with PML and is used mostly to maintain remission in moderate to severe CD with only modest effectiveness at induction of remission [10, 57]. In contrast, the most recently approved agent, ustekinumab, an IL-12/IL-23 inhibitor, has been shown to be as effective as anti-TNF therapy at inducing and maintaining remission in moderate to severe CD [10, 58].

Ultimately, the goal of medical therapy is to induce and maintain a steroid-free clinical remission, prevent complications and surgery, and improve the patient’s quality of life [10, 59]. For typical medication, complications, and monitoring recommendations, *see* Table 3.

A significant number of patients with CD can be managed by adopting a conservative approach that includes adequate rest, a nutritious diet, multivitamins, iron, folic acid, antioxidants, and sulfasalazine. Surgical therapy is useful in refractory disease and when complications such as occlusion, abscess, and fistulas develop. Though surgery is required to relieve obstruction, to repair a perforation, to treat an abscess, or to close a fistula, a judicious approach to treating the patient is of utmost importance regarding the decision whether to intervene or to continue with conservative management to avoid life-threatening complications [2]. This evidence confirmed the role of surgical intervention as reported in the index patient.

The outcome of CD has improved with good medical care. It is serious, but it is not a terminal illness. Mortality in these patients is due to risks of surgery or associated diseases [60]. These patients require very regular follow-up even if they are well, and any new symptom should be given due consideration. The index patient received adjuvant chemotherapy consisting of AZA, MTX, mesalamine, and methylprednisolone. He had complete disease remission.

With respect to prognosis, the literature suggests that almost all patients with CD have complications; perianal disease is present in approximately 50%. Approximately 40% will develop active disease within the first 3 years, and disease remains inactive over time in only a small percentage [14, 61]. The majority will require bowel resections and several surgeries. A review showed that 10 years after diagnosis, 85% had the same disease location; however, the initial pattern will change after 25 years [14, 61]. Stenosis or penetrating complications will be found in 60% of patients in the first 5 years, which will require intensive medical treatment (immunomodulatory and/or biological therapy) [14, 61].

The most recent postoperative abdominal CT scan and colonoscopy revealed disease-free status in the index patient. The index patient is currently receiving a maintenance dose of rectal mesalamine and oral omeprazole treatment. He has been followed every 2 months in the surgical outpatient clinic over the last 16 months with a satisfactory clinical outcome.

## Conclusions

CD is considered by many as a very rare disease in Africa. It is interesting to know that CD, which affects mainly young adults, may debut at any age. The symptoms of CD may mimic many other abdominal conditions for which medical attention is required. However, it should be kept in mind as one of the causes of acute abdomen, especially in those patients who have a long history of intestinal pathologies whose treatments greatly differ. Establishing an appropriate treatment in order to avoid short- and long-term complications, which may be life-threatening, depends mainly on distinguishing between other inflammatory disorders of the digestive tract and CD. A histopathologically confirmed diagnosis becomes very necessary also because of the emerging evidence that there is an increased risk of adenocarcinoma in patients with CD.
